# Early predictors for maltreatment-related injuries in infancy and long-term mortality: a population-based study

**DOI:** 10.1186/s12889-023-17180-8

**Published:** 2023-11-13

**Authors:** Hsin-Hung Chen, I-An Wang, Tan-Wen Hsieh, Jen-Huoy Tsay, Chuan-Yu Chen

**Affiliations:** 1 Institute of Public Health, National Yang Ming Chiao Tung University, Medical Building II, No. 155, Sec. 2, Linong Street, Taipei, 112 Taiwan; 2 Division of Pediatric Neurosurgery, The Neurological Institute, Taipei Veterans General Hospital, Taipei, Taiwan; 3 Center of Neuropsychiatric Research, National Health Research Institutes, Zhunan, Taiwan; 4 Department of Social Work, National Taiwan University, Taipei, Taiwan

**Keywords:** Infants, Child maltreatment, Death, Assaults

## Abstract

**Introduction:**

Incidence, health consequences, and social burden associated with child maltreatment appeared to be borne disproportionately by very young children. We conducted a population-based data linkage study to explore child- and family-level factors that affect receiving different diagnoses of maltreatment injuries and investigate excessive mortality throughout toddlerhood.

**Methods:**

We conducted a retrospective cohort study comprising 2.2 million infants born in 2004–2014 in Taiwan. Incident cases of child maltreatment were defined by hospitalization or emergency department visits for three heterogeneous diagnostic groups of maltreatment-related injuries (i.e., maltreatment syndrome, assaults, and undetermined causes) within 12 months after birth. The generalized linear model and landmark survival analyses were used to evaluate risk factors.

**Results:**

An estimated 2.9‰ of infants experienced at least one maltreatment-related injury, with a three-year mortality rate of 1.3%. Low birthweight was associated with increased risk of receiving the diagnosis of three maltreatment injuries, particularly maltreatment syndrome (adjusted Incidence Rate Ratio [aIRR] = 4.08, 95% confidence interval [CI]: 2.93–5.68). Socially advantaged family condition was inversely linked with receiving the diagnosis of maltreatment syndrome and assaults (e.g., high income: aIRR = 0.55 and 0.47), yet positively linked with undetermined cause (aIRR = 2.05, 95% CI: 1.89–2.23). For infants exposed to maltreatment, low birth weight and non-attendance of postnatal care were highly predictive of fatality; low birthweight served as a vital predictor for premature death during toddlerhood (aIRR = 6.17, 95% CI: 2.36–15.4).

**Conclusions:**

Raising awareness of maltreatment-related injuries in infancy and predictors should be a priority for appropriate follow-up assessment and timely intervention.

**Supplementary Information:**

The online version contains supplementary material available at 10.1186/s12889-023-17180-8.

## Background

Child maltreatment has emerged as a challenging issue for pediatric care providers and child protection practitioners worldwide. A recent systematic review of multinational surveys from 96 countries indicated that half of children aged 2–17 years experienced some form of violence each year globally, with an estimated 68% in Asia [1]. Data from national child protective service records in the United States showed that the cumulative probabilities were 32% for receiving at least one child maltreatment report by age 12 and 10% for substantiation [2]. Maltreatment experiences in early life have wide-ranging long-term negative consequences on individuals in later life stages, including health-compromising behaviors, physical illness, mental disorders, and social maladjustment, all of which may have a cascading burden on families and society [3–6]. To provide insight into the implementation of interventional strategies, it is imperative to characterize at-risk subgroups, identify early signs or symptoms, and devise timely and effective interventions [5].

Cumulative evidence indicated that incidence, health consequences, and social burden associated with child maltreatment were disproportionately borne by very young children [5, 7]. The annual incidence of hospitalization with physical severe abuse was 58.2 per 100,000 for those under one year of age, an estimated 12–20 times higher than that among 3 ~ 18 year-olds in the United States [8]; for abusive head trauma in Taiwan, the incidence among infants was nearly 20-fold higher than those preschoolers [9]. The studies involving maltreatment cases in child protective services also confirmed a similar high victimization rate before the first birthday [8, 10]. In 2020, an estimated 15% of maltreated victims were younger than one year at an incidence rate of 25.1 per 1,000, which was two-fold higher than the corresponding rates of 11.2, 10.4, and 9.7 per 1,000 in children aged 1, 2 and 3 years, respectively [11]. Notably, approximately one-half of fatal maltreatment occurs before one year old.

Clinically, identifying child maltreatment is challenging because the diagnosis relies on an array of clinical features, and evaluation often requires collaborative efforts [12, 13]. In the United States, studies evaluating hospital databases by child abuse pediatricians revealed that, despite high specificity, the sensitivity of International Classification of Diseases codes for child physical abuse was relatively low (54%~84%) [14, 15]. The diagnosis can be even more difficult for infants due to absence of available history from the victims, a lack of the witness, and the primary reliance on the caretakers’ report. To address this concern, when healthcare data were utilized as a source to monitor the occurrence of child maltreatment in the population, several clinical manifestations of injuries other than maltreatment syndrome were included as proxies. For instance, injuries related to physical assault in young children are often the result of parental or caregiver violence or poor supervision, and physical assault in infants has been indicative of neglect [16, 17]. To date, several studies have demonstrated potential contribution of individual- and family-risk factors for infant maltreatment (e.g., health condition and public insurance) [18–22]; nevertheless, few have systematically investigated whether such risk factors may differ by clinical manifestation of infant maltreatment [12, 23, 24]. In addition, prior investigations on the prognosis of abused infants were often limited to the index hospitalization period (i.e., fatal maltreatment), and prospective evidence concerning long-term mortality in surviving infants and its predictors is generally lacking.

In the present study, we focused on three distinct injuries of child maltreatment, with the overarching aim to examine the extent to which family- and child-level characteristics may differentially affect the diagnosis associated with child maltreatment taking place before the first birthday and to evaluate the factors accounting for excess death in maltreated infants throughout toddlerhood.

## Methods

### Data source

The present study used data from the 2004 to 2015 National Health Insurance (NHI) program, birth registration (BR), household registration (HR), and 2004–2018 death registration (DR) (see Supplementary Materials). The NHI is a mandatory, single-payer health insurance for all citizens and legal residents in Taiwan, providing healthcare in outpatient, ambulatory, and inpatient service systems from the first day of life since ﻿1995 [25]. The estimated coverage rate reached 99.5% of the population by the end of 2005 and 99.9% in 2018. In addition, the BR, HR, and DR datasets were used to obtain maternal sociodemographic information and to confirm the index child’s survival status. Based on the encrypted identification number for each enrolled individual, study variables in the study were linked and retrieved. This study protocol was reviewed and approved by the Institutional Review Board of National Health Research Institutes (EC1060510-E & EC1040910).

In this retrospective cohort study, we initially limited our study subjects to those born between January 1, 2004, and December 31, 2014 (n = 2,206,441) in Taiwan. To avoid duplication resulting from the episode-based inter-hospital referral for the same case (e.g., from a regional hospital to a medical center), we focused on the medical records for only the first maltreatment episode (i.e., as a proxy of the index incident episode).

### Measures

#### Indicators for infant maltreatment

The present study has ascertained infant maltreatment via maltreatment-related injury and marker condition [17, 26]. For maltreatment-related injury, hospitalization and/or emergency department visits in the first year of life were defined by the International Classification of Diseases, Ninth Revision, Clinical Modification (ICD-9-CM): maltreatment syndrome (ICD-9-CM codes 995·5, E967, 994·2, or 994·3), assaults (ICD-9-CM codes E960–E966, E968, E969; a proxy for inadequate supervision), and undetermined causes (ICD-9-CM codes E980–E989, V68·2, V70·4, V71·4, V71·5, V71·6, V71·81; a proxy for physical abuse or neglect) [17]. Maltreatment syndrome reflects physical abuse or neglect as the cause of injury. Assaults indicate assault by a caregiver (physical abuse) or violence by others, possibly due to inadequate supervision (neglect). Undetermined causes represent explicit uncertainty about the cause of the injury, which is a proxy for under-detected forms of physical abuse or neglect.

#### Survival status

For infants with any positive record of child maltreatment injury (n = 6297), the occurrence of death in the following three months and three years after the index hospital visit for infant maltreatment was confirmed by the national death registration. For comparative purposes, deaths among non-maltreated infants who survived beyond the age of six months (i.e., the average age of hospital visits for maltreatment) were obtained. Infant abuse-related severe injuries are recognized as “catastrophic illnesses,” for which co-payment will be exempted for treatment and prolonged hospitalization.

#### Child and family characteristics

For each child, information concerning infant-level characteristics (i.e., birth year, gender, and birthweight) was retrieved from the household registration records. Birthweight was categorized into three subgroups (i.e., ≥ 2500 g, 2000–2499 g, and < 2000 g). Family characteristics included family structure (i.e., marital status and having a child born three years before the index child) and socioeconomic condition (e.g., income, educational attainment, and the age of having the index child). Given that (i) the mother is often the primary caregiver in the study population and (ii) children are more likely to be linked with their mothers (i.e., mothers as insurance enrollees), we systematically retrieved socioeconomic characteristics from maternal records. Since the national health insurance premium is calculated mainly by the monthly income of insurance enrollees (e.g., children’s working mothers) and enrollee category (e.g., low-income households), we used premium as a proxy for family income, subgrouping children into high-income, middle-income, and low-income/poverty [27]. Maternal history of mental disorders in the three years preceding the birth of the index child included depressive disorders (ICD-9 CM codes of 290, 293–302, or 306–319) and substance use problems (ICD-9 CM codes of 291, 303, 304, 304·4, 304·7, 305·0, 305·2, 305·5, 357·5, 425·5, 535·30, 535·31, 571·0, 571·1, 571·2, 571·3, 965, 968·3, E850·0, E860·0, E935·0, or E938·3). Finally, the utilization of well childcare within the first two months after birth was used as a proxy for access to preventive care.

### Statistical analysis

Cross-tabulation was first conducted to analyze the distribution of child- and family-level characteristics, together with infant maltreatment and death. Given that the occurrence of diagnosed infant maltreatment injury was rare, the present study turned to the generalized linear models in zero-inflated Poisson distribution with robust variance estimation to evaluate the association estimates for possible predictors. The incidence in person-months was calculated for infants using the number of births for each study month as the denominator. The follow-up period ended either on the first birthday, date of death, or treatment seeking for probable infant maltreatment, whichever came first. The incidence rate ratio (IRR) and adjusted incidence rate ratio (aIRR) for the occurrence of the first diagnosis were the ratio of incident infant maltreatment divided by the total person-month at-risk. The 95% confidence intervals (CI) for the association estimates were also provided to facilitate interpretation.

Regarding the investigation of the prognostic factors for fatal maltreatment and long-term mortality, considering that the hazard function fluctuated over the course of a three-year follow-up and the survival probability was primarily conditioned on the length of index treatment (see Supplementary Materials), we decided to perform the landmark survival analyses to estimate the hazard ratio and 95% confidence interval of death for unbiased prediction. Here, the 90th day after the index treatment seeking for maltreatment was set as the landmark time: person-days of follow-up were first calculated from the index date of hospital visits to the date of death or the end of observation (i.e., 90th days) for fatal infant maltreatment. Next, to avoid immortal time bias, for those who survived after the 90th day (i.e., non-fatal maltreated infants), person-days of follow-up were calculated from the 91st day to the date of death or the end of observation (i.e., 1095th day). All data preparation and statistical analyses were performed using SAS 9·4 (SAS Institute, Cary, NC, USA).

## Results

Nearly 7% of the infants born between 2004 and 2014 weighed less than 2500 g at birth (see Table 1). Approximately one-quarter were enrolled with a low or subsided premium (i.e., low income/poverty), 16% had maternal educational attainment of junior high school or below, and 19% had never visited the well-child service within the first two months of life. The cumulative incidence of hospital visits for maltreatment-related injuries before the first birthday was 2.85 per thousand, with the undetermined cause being the majority. The 3-month and 3-year cumulative mortality rates after the index maltreatment injury were 0.79% and 1.3%, respectively, and the corresponding 3-year estimate in the comparison population (i.e., the birth year-matched children without maltreatment injuries, following up from 6 months old to 3·5 years old, n = 2157989) was 0.1%.

**Table 1:** Child and family characteristics for infants born in Taiwan in 2004–2014 (n = 2,206,441)

Variable	N	%
Birth year		
2004–2009	1,206,470	54.7
2010–2014	999,971	45.3
Gender		
Female	1,057,017	47.9
Male	1,149,424	52.1
Birthweight (g)^a^		
≥ 2500	1,950,802	88.4
2000–2499	123,595	5.6
< 2000	34,357	1.6
Maternal marital status^b^		
Married	1,952,262	88.5
Single	84,403	3.8
Others (e.g., divorced and widowed)	43,480	2.0
Missing	237,407	10.8
Having at least one older child^c^		
No	1,632,757	74.0
Yes	573,684	26.0
Income level (insurance status)^d^		
Low income/poverty	542,842	24.6
Middle income	1,121,927	50.9
High income	541,672	24.6
Maternal educational attainment^a^		
Junior high school or below	342,608	15.5
Senior high school	694,496	31.5
College or above	1,119,503	50.7
Maternal age at having the index child (years)^a^		
35 or above	41,168	1.9
20–34	1,776,026	80.5
< 20	341,394	15.5
Well-child care utilization in the first two months of life^d^		
Yes	1,793,892	81.3
No	412,549	18.7
Maternal depression ^d,e^		
No	2,194,753	99.5
Yes	11,688	0.5
Maternal substance use-related problems ^d,e^		
No	2,205,747	99.97
Yes	694	0.03
ICD-9-CM (per 1,000)^d^		
Maltreatment-related injuries^f^	6297	2.85
Maltreatment syndrome	579	0.26
Assaults	166	0.08
Undetermined causes	5580	2.52
Death within three months of index visits (per 1,000)^g^	50	7.94
Death within three years of follow-up (per 100)^g^	79	1.25

Note. Columns may not add up to 100% due to missingness

a. Retrieved from the Birth Registry in the Ministry of the Interior

b. Retrieved from the Household Registration of the Ministry of the Interior

c. Older child: within three years of the index child’s birth

d. Retrieved from the National Health Insurance Research Database of the National Health Insurance Administration

e. During three years before the index delivery

f. Maltreatment syndrome: ICD9 codes 995·5 (n = 536), E967, 994·2, and 994·3; assaults: ICD9 codes E960, E961, E962, E963, E964, E965, E966, E968, and E96; undetermined causes: ICD9 codes E980, E981, E982, E983, E984, E985, E986, E987, E988, E989, V68·2, V70·4, V71·4, V71·5, V71·6, and V71·81

g. Retrieved from the Death Registration

For the occurrence of hospital visits for the three clinical manifestations of child maltreatment injury, our analyses indicated that low birthweight was the most consistent child-level predictor (see Table 2). With statistical adjustment for listed characteristics, having a birthweight of less than 2000 g may increase the risk of maltreatment syndrome, assaults, and undetermined causes by 308%, 133%, and 98%, respectively. As to family socioeconomic status, the risks varied by clinical manifestation of maltreatment: disadvantaged family characteristics were unanimously associated with increased risk of maltreatment syndrome and assault (e.g., low maternal education for maltreatment syndrome: IRR = 2.76; 95% CI: 2.16–3.52), whereas advantaged family characteristics were strongly linked with receiving undetermined cause maltreatment-related injuries (e.g., high income: IRR = 2.05; 95% CI: 1.89–2.23). As to maternal health conditions, the increased risk linked with depression and substance use problems was only found in maltreatment syndrome (aIRR = 1.57 and 2.42).

**Table 2:** Child and family characteristics associated with hospital visit for maltreatment-related injury in infancy (n = 2,206,441)

Variable	Maltreatment-related injury		
	Maltreatment syndrome aIRR (95% CI)	Assaults aIRR (95% CI)	Undetermined cause aIRR (95% CI)
Birth year (Ref: 2004–2009)			
2010–2014	1.18 (1.00, 1.39)^*^	0.98 (0.72, 1.33)	1.66 (1.57, 1.75)^***^
Age (Ref: 8–11 months old)			
4–7	1.57 (1.26, 1.96)^***^	0.80 (0.57, 1.11)	2.61 (2.38, 2.86)^***^
0–3	2.00 (1.62, 2.47)^***^	0.39 (0.25, 0.58)^***^	5.39 (4.96, 5.86)^***^
Gender (Ref: Female)			
Male	1.36 (1.16, 1.61)^***^	1.29 (0.95, 1.74)	0.99 (0.94, 1.05)
Birthweight (Ref: ≥2500 g)^a^			
2000–2499	1.83 (1.40, 2.39)^***^	0.81 (0.40, 1.66)	1.10 (0.98, 1.23)
<2000	4.08 (2.93, 5.68)^***^	2.33 (1.03, 5.30)^*^	1.98 (1.69, 2.31)^***^
Maternal marital status (Ref: Married)^b^			
Single	2.68 (2.08, 3.45)^***^	1.36 (0.72, 2.58)	1.16 (1.01, 1.34)^*^
Others (divorced, widowed)	2.40 (1.74, 3.32)^***^	0.91 (0.33, 2.51)	0.87 (0.68, 1.11)
Missing	0.47 (0.32, 0.69)^***^	0.48 (0.24, 0.95)^*^	1.00 (0.88, 1.13)
Having at least one older child (Ref: No) ^c,d^			
Yes	1.32 (1.10, 1.58)^**^	0.96 (0.68, 1.35)	0.88 (0.83, 0.94)^***^
Income level (insurance status) (Ref: Low income/poverty) ^c^			
Middle income	0.56 (0.47, 0.67)^***^	1.02 (0.72, 1.44)	1.12 (1.04, 1.21)^**^
High income	0.55 (0.42, 0.72)^***^	0.47 (0.28, 0.80)^**^	2.05 (1.89, 2.23)^***^
Maternal educational attainment (Ref: College or above)^a^			
Senior high school	1.73 (1.39, 2.14)^***^	1.03 (0.72, 1.47)	0.59 (0.55, 0.63)^***^
Junior high school or below	2.76 (2.16, 3.52)^***^	1.64 (1.05, 2.55)^*^	0.47 (0.42, 0.52)^***^
Maternal age at having the index child (Ref: 35 years old or above)^a^			
20–34	0.98 (0.77, 1.24)	1.55 (0.93, 2.57)	0.70 (0.66, 0.75)^***^
<20	1.61 (1.08, 2.40)^*^	1.35 (0.47, 3.87)	0.36 (0.24, 0.54)^***^
Maternal depression (Ref: No) ^c,e^			
Yes	1.57 (1.08, 2.27)^*^	1.36 (0.60, 3.09)	1.06 (0.89, 1.27)
Maternal substance use problems (Ref: No)^c,e^			
Yes	2.42 (1.45, 4.04)^***^	0.78 (0.11, 5.61)	0.82 (0.53, 1.26)

Note. Crude incidence rate ratio (cIRR); adjusted incidence rate ratio (aIRR) estimates were obtained via the Age-Period-Cohort Model;

* p < 0.05, ** p < 0.01, *** p < 0.001

a. Retrieved from the Birth Registry in the Ministry of the Interior

b. Retrieved from the Household Registration of the Ministry of the Interior

c. Retrieved from the National Health Insurance Research Database of the National Health Insurance Administration

d. Older sibling: within 3 years of the index child

e. During three years before the index delivery

For those experiencing maltreatment-related injury, low birthweight and maltreatment type appeared to be the strongest predictors for fatality and long-term mortality in those experiencing maltreatment-related injuries (see Table 3). Compared with those with undetermined causes of maltreatment injury, infants with maltreatment syndrome had a 16.7-fold hazard of death within three years of the index visit (95% CI: 6.60–42.1). Additionally, not utilizing postnatal services was associated with increased risk of death within three months of maltreatment-related injuries (aHR = 2.24).

**Table 3:** Child and family characteristics predicting fatal maltreatment and long-term mortality in the maltreated infants (n = 6295)

Variable	Fatal maltreatment		Three-year survival status	
	cHR (95% CI)	aHR (95% CI)	cHR (95% CI)	aHR (95% CI)
Birth year (Ref: 2004–2009)				
2010–2014	0.52 (0.29, 0.91)^*^	0.87 (0.49, 1.55)	0.87 (0.42, 1.82)	
Gender (Ref: Female)				
Male	1.15 (0.66, 2.01)		1.72 (0.80, 3.70)	
Birthweight (Ref: ≥2500 g)^a^				
2000–2499	3.47 (1.60, 7.52)^**^	1.69 (0.77, 3.75)	2.33 (0.69, 7.91)	1.49 (0.43, 5.16)
< 2000	6.74 (3.10, 14.6)^***^	2.84 (1.26, 6.38)^*^	10.8 (4.52, 25.9)^***^	6.17 (2.46, 15.4)^***^
Maternal marital status (Ref: Married)^b^				
Single	5.47 (2.61, 11.5)^***^	1.44 (0.62, 3.32)	3.61 (1.24, 10.5)^*^	1.50 (0.46, 4.87)
Others (divorced, widowed)	10.3 (4.56, 23.4)^***^	1.92 (0.78, 4.72)	2.20 (0.30, 16.3)	0.77 (0.10, 6.14)
Missing	0.73 (0.18, 3.04)	0.75 (0.18, 3.25)	1.06 (0.25, 4.52)	1.27 (0.28, 5.76)
Having at least one older child (Ref: No)^c,d^				
Yes	2.05 (1.17, 3.62)^*^	1.57 (0.86, 2.85)	2.18 (1.04, 4.57)^*^	1.64 (0.75, 3.58)
Income level (insurance status) (Ref: Low income/poverty)^c^				
Middle income	0.35 (0.20, 0.62)^***^	0.93 (0.49, 1.79)	0.58 (0.25, 1.32)	1.30 (0.52, 3.24)
High income	-	-	0.27 (0.10, 0.74)^*^	1.12 (0.33, 3.83)
Maternal educational attainment (Ref: College or above)^a^				
Senior high school	7.32 (3.48, 15.4)^***^	1.09 (0.49, 2.44)	3.21 (1.34, 7.72)^**^	1.36 (0.50, 3.71)
Junior high school or below	12.1 (5.55, 26.5)^***^	0.82 (0.33, 2.01)	6.55 (2.66, 16.1)^***^	1.49 (0.46, 4.78)
Maternal age at having the index child (Ref: 35 years old or above)^a^				
20–34	3.46 (1.24, 9.64)^*^	1.81 (0.64, 5.13)	1.16 (0.47, 2.87)	0.82 (0.32, 2.09)
< 20	20.7 (5.18, 82.7)^***^	1.66 (0.37, 7.41)	7.19 (1.45, 35.6)^*^	1.05 (0.17, 6.38)
Maternal depression (Ref: No)^c,e^				
Yes	0.78 (0.11, 5.62)		-	
Maternal substance use problems (Ref: No)^c,e^				
Yes	3.35 (0.46, 24.3)		-	
Well-child care utilization in the first two months of life (Ref: Yes)^c^				
No	6.48 (3.72, 11.3)^***^	2.24 (1.24, 4.05)^**^	2.74 (1.25, 6.02)^*^	1.19 (0.52, 2.76)
Maltreatment-related injuries (Ref: Undetermined cause only)^c^				
Maltreatment syndrome only	90.3 (35.8, 228.0)^***^	37.8 (13.8, 103.1)^***^	23.6 (10.7, 52.1)^***^	16.7 (6.60, 42.1)^***^
Assault only	7.57 (0.89, 64.7)	4.36 (0.50, 37.7)	-	-
Two or more	41.0 (4.79, 350.9)^***^	21.9 (2.51, 190.3)^**^	23.2 (2.93, 182.8)^**^	18.8 (2.29, 154.6)^**^

Note. HR: Hazard Ratio; obtained via Cox regression model; aHR: Adjusted Hazard Ratio. * p < 0.05, ** p < 0.01, *** p < 0.001

a. Retrieved from the Birth Registry in the Ministry of the Interior

b. Retrieved from the Household Registration of the Ministry of the Interior

c. Retrieved from the National Health Insurance Research Database of the National Health Insurance Administration

d. Older sibling: within three years of the index child

e. During three years before the index delivery

## Discussion

To our knowledge, this is the first population-based study to investigate the incidence of child maltreatment-related injury in the first year of life, long-term mortality, and its predictors in the Asian region. Our findings can be summarized as follows: First, nearly 3 in 1000 children born in Taiwan experienced a maltreatment-related injury in the first year of life, with three-year mortality rate of 1.3%. Second, low birthweight served as the strongest child-level risk factor for the incidence of three types of child maltreatment. Socially advantaged family condition was linked with reduced risk of receiving the diagnosis of maltreatment syndrome and assaults yet elevated risk of maltreatment-related injury with an undetermined cause. Finally, low birthweight and maltreatment type appeared to be important predictors of long-term mortality rates.

In developed countries, there is wide variation in the incidence of maltreatment-related injuries in infants [17, 28]. Our estimate of maltreatment syndrome and assaults generally fell within the range of lower bounds (only higher than 11.5/100,000 in Sweden) [17]. Given the higher rate of undetermined causes (as compared with maltreatment syndrome and assaults), we cannot rule out the possibility that the present estimates of maltreatment syndrome (ICD 999.5) were still underestimated, with possible explanations including (i) inadequate, unclear, or incomplete information to code “child maltreatment,” (ii) lack of relevant training to collect medical history or the inability/unwillingness to arrange appropriate examination (e.g., as a result of the constrained reimbursement), and (iii) the reluctance of healthcare professionals to report suspected abuse or get involved in the joint investigation [26].

In our study, unfavorable birth conditions (i.e., low birthweight) were not only associated with an increased risk of maltreatment-related injury in infants [20, 21, 29–32] but also linked with an excessive risk of maltreatment fatality and subsequent premature death [33]. One possible explanation for the finding would be that taking care of an infant with low birthweight may add an extra burden to already strained parents, escalating psychological distress or provoking hostile parental feelings, consequently leading to physical abuse or neglect [34]. An alternative explanation is that infants with low birthweight were more vulnerable to unskilled parenting practices or disadvantaged social environment, consequently exacerbating the severity of maltreatment, assault, and injury [35]. Selective prevention measures targeting caregivers should consider integrate the elements such as parenting education and respite and selfcare. Finally, it is plausible that there is a shared pathway (or unmeasured risk factor) underlying low birthweight, maltreatment-related clinical manifestations, and subsequent premature death, such as inadequate nutrition, congenital metabolic disorders, unsafe housing conditions, or domestic violence [36].

Disadvantaged socioeconomic status has been long indicative of increased maltreatment-related hospitalization or death in infants [20–22, 37–39]. In our study, the alarmingly elevated risk of a severe form of maltreatment-related injury (i.e., maltreatment syndrome) in association with disadvantaged socioeconomic status may be affected via processes like differential parenting practice, caregiver features, household composition or context (e.g., unrelated adults), neighborhood milieu, treatment-seeking behaviors, and other factors [21, 22, 37–40]. Together with maternal history of depression or substance use problem-related risk [32], our findings may justify the implementation of interventions to address the social needs of pregnant women in prenatal and perinatal healthcare [5]. However, we also noticed that the association estimates were generally opposite for maltreatment injuries with undetermined causes. Prior evidence indicates that socioeconomic status may affect the diagnostic procedure or outcome [41, 42]. As such, we cannot rule out the possibility that this observation was partly due to, at least in part, that physicians or other healthcare professionals were more likely to suspect child abuse when the socioeconomically disadvantaged infants were presented with unwitnessed or unknown injuries. Given that neither screening guidelines nor standardized clinical pathways exist for evaluating the infants with injuries in Taiwan, future research that takes socioeconomic profiles into account is needed, especially when conflicting, unconvincing, or no explanations are provided [43, 44].

In Taiwan, the well-childcare has been fully covered by governmental funds, providing a comprehensive assessment of a child’s growth, developmental check-up, and vaccination. Extending prior studies that indicated under-immunization was a risk factor for child maltreatment in young children [45], our analyses revealed that no utilization of well-child care appeared to be a strong predictor for severe maltreatment. Although the reasons for not attending well-child service cannot be determined from this healthcare claims data-based analysis, it is possible that the underlying family and child factors may also pose threats to health and development of maltreated infants presumably in need of intensive attention or heightened monitoring.

Some potential limitations of this study should be considered. First, since the database is designed for reimbursement purposes, the validity and completeness of coding for child maltreatment (ICD 9 CM-code:995·5) in the NHI claims data may not be satisfactory [24, 46]. Further validation of the subsample for each maltreatment measure may be necessary to improve the utility of claims data as a passive surveillance system to safeguard children’s safety and well-being. Second, data were not available concerning some known risk factors for child abuse, particularly maternal smoking during pregnancy, paternal substance abuse, and contact history with child protective services. These and other unmeasured characteristics may partially account for the observed disadvantaged socioeconomic condition and low birthweight-related risk of incident child maltreatment and subsequent premature death (e.g., congenital, or other postnatal diseases). Third, our follow-up analyses of maltreated infants did not capture information on the cause of death. Additionally, time-varying hospital visits and health conditions after the index maltreatment episode were not considered in survival analyses. It is possible that the maltreated infants were discharged with sequelae or later readmitted with maltreatment-related injury. Nevertheless, these variables may play the role of mediators operating between maltreatment and death.

Notwithstanding these limitations and methodological issues, our findings are an important addition to the literature because only a few population-based studies have documented the incidence of child maltreatment in the first year of life, especially in regions where the economic burden of child maltreatment is substantial [47]. Second, since the prevalence of “diagnosed” child maltreatment is relatively uncommon, the 11-year large sample (> 2 million) enabled us to systematically investigate and meticulously compare the predictors in different clinical manifestations of probable infant maltreatment with reasonable comprehensiveness. Finally, the study has utilized the prospective nature of evidence to advance our understanding toward differential effects of unfavorable perinatal outcomes and family socioeconomic conditions on the prognosis of suspected maltreatment from a long-term perspective.

## Conclusions

Our observed findings with respect to the predictors for maltreatment injury and subsequent excess death may be worthy of future attention in relation to preventive implications in infant maltreatment in the context of healthcare system [48, 49]. Evidence-based intervention strategies — such as increasing clinicians’ awareness and understanding of different clinical manifestations of physical abuse in infancy and implementing a standardized tool/clinical pathway to screen for young children with severe trauma and injury— may enable healthcare providers to reduce the sole reliance (or bias) on the sociodemographic category to make a decision [42].

### Electronic supplementary material

Below is the link to the electronic supplementary material.

Supplementary Material 1

## Data Availability

The data supporting the findings are available from the Department of Statistics of Taiwan Ministry of Health and Welfare (MOHW) upon the application. All the data were stored in the Health and Welfare Data Science Center (HWDC). The availability of these data is not publicly available due to the legal restriction. If someone wants to request the information regarding data from this study, please contact chuanychen@nycu.edu.tw.

## Abbreviations

aIRR: Adjusted Incidence Rate Ratio
BR: Birth Registration
DR: Death Registration
HR: Hazard Ratio
HR: Household Registration
IRR: Incidence Rate Ratio
ICD-9-CM: International Classification of Diseases, Ninth Revision, Clinical Modification
NHI: National Health Insurance
