# Supplementary material for: Early predictors for maltreatment-related injuries in infancy and long-term mortality: a population-based study
Source: BMC Public Health. 2023 Nov 13;23:2232. doi: 10.1186/s12889-023-17180-8 (PMC10641954; doi:10.1186/s12889-023-17180-8)
Supplement: Supplementary file 1 — Supplementary Material 1 [file 12889_2023_17180_MOESM1_ESM.docx]

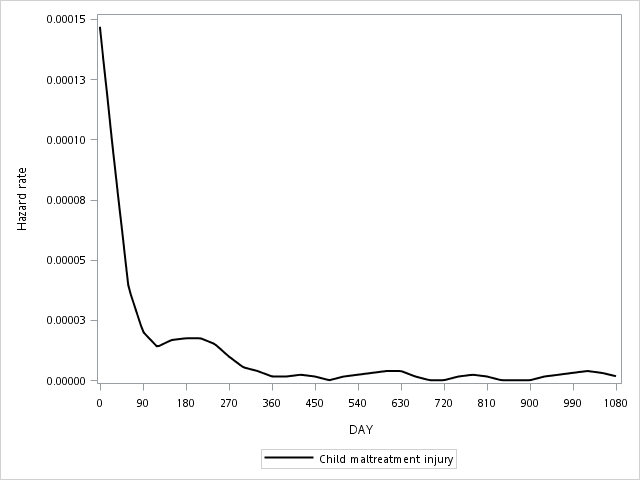


**Figure S1*.*** The hazard rate of death in the three years of the index hospital visit for child maltreatment^a^

1. Within the year following hospital visit for infant maltreatment. The hazard of death rapidly declined during the third month (i.e., 60th–90th day) after the index hospital visit.

# Table S1. List of the datasets

| **The Name of the Dataset** | **Covered Year** | **Variables** |  |
| --- | --- | --- | --- |
| **The Ministry of the Interior (MOI)** | | | |
| Household Registration (HR) | 2004-2015 | Maternal birth year, educational attainment, and marital status.  # of young children in the household |  |
| Birth Registration (BR) | 2004-2014 | Date of birth, birth weight, gender |  |
| Death Registration (DR) | 2004-2018 | Date of death |  |
| **National Health Insurance Research Database (NHIRD) from the MOHW** | | | |
| Registry for Beneficiaries | 2004-2015 | Insurance premium (i.e., income status) |  |
| Ambulatory Care Expenditures by Visits | 2004-2015 | Emergency room visit |  |
| Inpatient Expenditures by Admissions | 2004-2015 | Hospitalization records |  |
